# Bovine Milk Extracellular Vesicles Modulate Alveolar Bone Microarchitecture and Mitigate Hepatic Steatosis in Obese Mice Fed a High‐Fat Diet

**DOI:** 10.1002/mnfr.70400

**Published:** 2026-02-05

**Authors:** Francine R. F. Silva, Joyce E. Heredia, Bruna C. Oliveira, Onno J. Arntz, Talita Martins, Eduardo H. M. Nunes, Mauro M. Teixeira, Tarcilia A. Silva, Fons A. J. van de Loo, Soraia Macari, Adaliene V. M. Ferreira, Marina C. Oliveira

**Affiliations:** ^1^ Immunometabolism, Department of Nutrition, Nursing School Universidade Federal de Minas Gerais Belo Horizonte Brazil; ^2^ Experimental Rheumatology Radboud University Medical Center Nijmegen the Netherlands; ^3^ Department of Metallurgical and Materials Engineering School of Engineering Universidade Federal de Minas Gerais Belo Horizonte Brazil; ^4^ Immunopharmacology, Department of Biochemistry and Immunology Institute of Biological Sciences Universidade Federal de Minas Gerais Belo Horizonte Brazil; ^5^ Department of Oral Surgery and Pathology Faculty of Dentistry Universidade Federal de Minas Gerais Belo Horizonte Brazil; ^6^ Department of Restorative Dentistry Faculty of Dentistry Universidade Federal de Minas Gerais Belo Horizonte Brazil

**Keywords:** adipose tissue, bone loss, extracellular vesicles, liver, milk, obesity

## Abstract

Obesity is linked to low‐grade inflammation and systemic bone loss. Current treatments are limited, necessitating new therapeutic approaches. Bovine milk extracellular vesicles (MEVs) modulate bone cell activity, although their role in bone during diet‐induced obesity is unexplored. We evaluated MEV influence on metabolism and bone in a model of high‐fat (HF) diet‐induced obesity. C57BL/6 mice were fed with a control (C) or an obesogenic diet for 12 weeks, with MEV treatment in drinking water starting in the 9th week. HF diet‐fed mice showed loss in the alveolar bone and femur, characterized by a reduced number of osteoblasts, osteocytes, and an increase in osteoclasts. Augmented adiposity and liver fat deposition were found, correlating with hyperglycemia and hyperlipidemia. MEV treatment improved alveolar bone parameters along with a positive balance between osteocytes and osteoblasts versus osteoclast populations. MEVs did not change femur parameters, but reduced osteoclasts. MEVs did not modify systemic metabolism or adipose tissue morphology, but they reduced hepatic fat accumulation. HF diet induces bone loss and metabolic changes. MEV treatment exerts a local cellular effect on alveolar bone, but cannot reverse HF‐induced bone loss in the femur. Nevertheless, MEVs demonstrate benefits in reducing liver fat accumulation.

## Graphical abstract

Obesity induced by an HF diet led to increased adiposity, bone loss in the femur and maxilla, metabolic changes, and fatty liver in mice. Treatment with MEVs did not alter adiposity, metabolic changes, or femoral bone loss, but reduced alveolar bone loss and fat deposition in the liver of obese mice. Created in BioRender. Ferreira, A. (2025) https://BioRender.com/k3zhm7i.

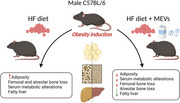

**Abbreviations**
ABC‐CEJ: alveolar bone crest and the cemento‐enamel junction
BMD: bone mineral density
BV/TV: bone volume per total tissue volume
EAT: epididymal adipose tissue
HF: high‐fat
MEV: bovine milk extracellular vesicle
RMD: root mineral density
RV/TV: root volume per total tissue volume
SAT: subcutaneous adipose tissue
SMI: model structure index
Tb.N: number of trabeculae
Tb.Sp: trabecular separation
Tb.Th: trabecular thickness

## 1 Introduction

Obesity is a chronic disease characterized by adipose tissue accumulation associated with low‐grade inflammation and metabolic disorders such as osteoporosis [1, 2, 3]. Inflammatory mediators produced from hypertrophic adipocytes may negatively interfere with bone metabolism. By modifying the activity of bone cells and increasing tissue reabsorption over matrix deposition, these mediators contribute to the disruption of bone microarchitecture [4, 5, 6, 7]. Given the widespread nature of obesity, treatments that contribute to an overall improvement in metabolism are desired. Importantly, treatment for bone loss is primarily limited to medications and supplementation with calcium and vitamin D [8, 9, 10, 11], highlighting the need for new therapeutic strategies.

Recently, bovine MEVs have emerged as a promising component for maintaining bone health [12, 13, 14, 15, 16]. These naturally derived molecules are formed from the plasma membrane and contain proteins, lipids, and ribonucleic acids (RNA), including tRNA, mRNA, and miRNA, which, when transported via vesicles, can exert functions, elsewhere in the organism [17, 18]. Bone cells, such as osteoblasts and osteoclasts, are responsive to MEVs in vitro and in vivo [12, 16, 19, 20], demonstrating their potential to modulate bone cell activity and treat bone loss. Our research group previously showed that MEVs improve bone health and metabolism in mice fed a high‐refined carbohydrate diet, a mild obesity model exhibiting no alteration in body weight gain, but systemic and local metabolic alterations in adipose tissue and liver, alongside bone loss [12, 21]. However, we still do not know their effect in an obesity model induced by a HF diet, which is a standard model for inducing severe metabolic disorders, body weight gain, and bone loss.

We hypothesized that MEVs can influence the metabolism in obesity, contributing to bone homeostasis, possibly by directly modulating bone cells and favoring bone formation. Therefore, we aimed to evaluate whether treatment with these components affects cellular mechanisms underlying obesity‐induced bone loss, analyzing both femoral and alveolar bone to distinguish systemic from potential local effects. Additionally, we assessed whether the treatment impacts the systemic metabolic profile and specific organ changes, particularly in adipose tissue, and the liver, associated with HF diet‐induced obesity.

## 2 Materials and Methods

### 2.1 Animal Experimental Protocol

For 12 weeks, eight‐week‐old male C57BL/6 mice were maintained in a controlled environment with a 12 h light/dark cycle with ad libitum access to water and food. They were randomly divided into three groups (*n* = 6 for each group): i) Control (C)—consumption of standard AIN93 diet; ii) High‐fat diet (HF)—consumption of a diet rich in saturated fat; iii) HF‐MEVs—consumption of a diet rich in saturated fat + treatment with bovine MEVs. The control (C) group received the standard AIN93M diet, consisting of corn starch, maltodextrin, casein, sucrose, cellulose, soybean oil, AIN93M mineral, and vitamin mix, choline bitartrate, methionine, BHT, and distilled water. The HF diet was derived from the AIN93M standard diet but was composed of 45% fat (mainly lard) (Table S1). The nutritional information for each diet is shown in the Table S2. The, “UFMG Animal Experimentation Ethics Committee” (protocol number: 182/2023) approved the experiments. Treatment protocols regarding MEV dosage and administration are detailed in Section 2.2. The animals were fed the respective diets throughout the experimental period and weighed weekly to assess weight gain. After 12 weeks, the mice were euthanized by exsanguination following anesthesia with ketamine (80 mg/kg) and xylazine (10 mg/kg) diluted in 0.9% NaCl. Blood samples were collected and centrifuged at 3000 × g for 10 min at 4 °C to obtain serum, which was stored at ‐80 °C for biochemical analyses. The maxilla and femur were immediately fixed in 10% neutral buffered formalin for 48 h for micro‐CT and histological analyses. Liver and adipose tissue samples were weighed and a portion was fixed in 10% buffered formalin for histological assessment, while the remaining tissue was frozen and stored at ‐80 °C for lipid extraction and gene expression analysis, while the remaining adipose tissue was stored at ‐80 °C. The adiposity index was calculated according to the proximate proportion of the animal's adiposity in relation to body weight. It is calculated by the sum of the three visceral fat deposits, measured in the experiments (epididymal, retroperitoneal, and mesenteric adipose tissue), divided by the final weight of the animal, and this value multiplied by 100.

### 2.2 Bovine MEVs Isolation and Dosage Information

MEVs were obtained from the Experimental Rheumatology Laboratory (Radboudumc, Nijmegen, the Netherlands). MEVs were isolated from a single batch of semi‐skimmed commercial milk using a differential ultracentrifugation protocol previously validated by our group [16, 17]. Briefly, to eliminate cell debris, fat globules, and somatic cells, the milk underwent initial centrifugation at 70,000 × g for 1 h at 4 °C. Following this step, the supernatant was serially filtered through Whatman papers (No. 1 and No. 50) and subsequently passed through a 0.22 µm pore filter. The filtrate was then subjected to ultracentrifugation at 100,000 × g for 90 min at 4 °C to pellet the vesicles. The resulting pellets were resuspended in PBS and washed five times, followed by a final centrifugation step at 2000 × *g* for 10 min (4 °C) to remove potential contaminants. The supernatant containing the purified MEVs was sterilized using 0.22 µm syringe filters. Protein concentration was quantified using the Micro‐BCA assay (Thermo Scientific, Pierce, Rockford, IL). As previously described, particle characterization included proteomic analysis, identification of specific markers (e.g., CD63), miRNA profiling, and milk‐specific proteins [17, 22, 23]. Additional characterization was performed using Nanosight tracking analysis (NS300), and the MEVs had an average diameter of approximately 140 nm. According to the International Society of Extracellular Vesicles, MEVs have all the characteristics of exosomes [24].

MEVs were administered in the drinking water of animals in the treatment group at a dose of 14.3 × 10^6^/mL of particles (approximately 143 nanograms of protein/mL). This dosage was selected based on previous doseresponse studies demonstrating its efficacy in modulating bone metabolism without adverse effects in mice fed a chow diet [19, 20]. The treatment started in the nineth week, a time point where the obesity phenotype (including weight gain and metabolic dysregulation) is already established in this model [25]. This specific window was chosen to evaluate the potential of MEVs to counteract the progression of metabolic and skeletal complications associated with established obesity. Thus, the experimental design assesses a therapeutic intervention aimed at attenuating tissue damage rather than a prophylactic strategy to prevent the onset of metabolic disease. The administration continued for four weeks, until the end of the experiment (12 weeks). Based on an estimated water intake of 5 mL/day for a mouse and average body weight of 25 g, the daily dose corresponded to 28.6 µg of EV protein/kg of body weight (HEDs of 162 µg protein/day for a 70 kg person). We emphasize that the protein estimate for human consumption was used solely to provide context for the dosage and should not be interpreted as the primary dosage metric. The MEV vehicle (PBS) was administered at an equivalent volume dose in non‐treated mice in the same period. Drinking water was replaced every 2–3 days.

### 2.3 Micro‐Computed Tomography (micro‐CT)

Samples of the maxilla and femur were scanned using the Skyscan 1174 system (Skyscan, Aartselaar, Belgium) to evaluate bone microstructure. Image reconstruction was performed using NRecon software (Skyscan, Belgium), and 3D positioning was standardized for all samples using DataViewer (Skyscan, Belgium). Quantitative morphometric analysis was conducted with CTAn software (Skyscan, Belgium). Regions of interest (ROIs) were defined at the distal femoral metaphysis and the furcation area of the first upper maxillary molar. Key parameters analyzed included BMD, bone volume fraction (BV/TV), Tb.Th, trabecular number (Tb.N), Tb.Sp, and the structure model index (SMI). Cortical bone parameters (Ct.Ar, Ct.Th, and Ct.Ar/Tt.Ar) were also assessed in the femur. RMD and root volume per total tissue volume (RV/TV) were evaluated in all root extensions in the mesiobuccal, distobuccal, and palatal roots of first molars, with irregular anatomical regions of interest drawn manually [16, 26]. The height of alveolar bone crest loss was determined by measuring the distance between the alveolar bone crest (ABC) and the cementoenamel junction (CEJ), known as ABC‐CEJ in three‐dimensional images, from the mesial surface of the maxilla first molar to the distal surface of the maxillary third molar on the palatal surface using ImageJ software (National Institutes of Health, MD, USA).

### 2.4 Histomorphometric Analysis

The collected maxillae, and femurs were fixed with 4% buffered formalin solution for histological processing. Adipose tissues and liver were dehydrated, cleared, and embedded in paraffin. Five‐micrometer‐thick histological sections stained with hematoxylin‐eosin (H&E) were obtained to analyze the adipocyte area and liver histopathological score using ImageJ software (NIH Image, Bethesda, MD, United States). The bone samples underwent the decalcification process with EDTA and were impregnated with paraffin.

Histological images were captured using a digital camera (Canon PowerShot A620, Tokyo, Japan) coupled to a light microscope (Carl Zeiss, Göttingen, Germany). Quantitative analyses were conducted using ImageJ software (NIH, Bethesda, MD, USA). All histological analyses were performed under appropriate magnifications (e.g., 100× for adipocytes, 400× for bone cells, and liver histopathology). The number of bone lining osteoblasts was determined on Masson's Trichrome staining sections along the length of the alveolar bone and bone trabeculae of the femur in a given area. Osteocytes were quantified on H&E staining sections, considering the total number of lacunae in the alveolar bone and the femur. Osteocyte density was determined by normalizing the number of counted lacunae by the total bone area analyzed in the alveolar and femoral sections. Furthermore, osteoclasts were evaluated by a TRAP staining kit (Sigma‐Aldrich, St Louis, MO, USA). Counts of TRAP‐positive multinucleated osteoclasts were performed along the length of the alveolar bone in the furcation region of the first molar and the distal metaphysis of the femur [16, 21].

The cellular area of all adipocytes with a well‐defined cell border per animal was measured to assess the adipocyte size. The histopathological score evaluates the presence of hepatic steatosis, inflammatory infiltrate, and ballooning of hepatocytes. Each characteristic was scored based on the degree of alteration: 1) absent, 2) minimal presence, 3) moderate presence, and 4) marked presence. The final score was calculated as the sum of all values [27].

### 2.5 Oral Glucose Tolerance Test (OGTT)

After a 6 h fasting, mice received a D‐glucose solution (2 mg/g of body weight) through gavage. At 0, 15, 30, 60, and 120 min, peripheral blood glucose readings were performed using the Accu Chek advantage glucometer.

### 2.6 Serum Analyses

The bone metabolism markers RANKL and OPG, as well as metabolic adipose serum markers such as adiponectin and leptin, were quantified in mouse serum using DuoSet ELISA kits (R&D Systems Europe Ltd., Abington, United Kingdom). Serum concentrations of glucose, total cholesterol, triglycerides, and alanine aminotransferase (ALT), aspartate aminotransferase (AST), and gamma‐glutamyl transferase (GGT) were assessed using enzymatic kits (Bioclin, Belo Horizonte, MG, Brazil). Analyses were performed according to the manufacturer's instructions.

### 2.7 Measurement of Liver Lipid Content

Total lipids were extracted from the animals' liver according to the method of Folch et al. [28]. Briefly, liver tissue was homogenized with chloroform: methanol solution (2:1) to extract total fat from the sample. After removal, the solution was filtered and mixed with 0.9% saline. The upper phase was aspirated, and the lower phase aliquot was added to the pre‐weighed container. After evaporation, the obtained extract was mixed with 500 µL of isopropanol to evaluate total fat. Cholesterol and triglyceride levels were then assessed using enzymatic kits (Bioclin, Belo Horizonte, MG, Brazil).

### 2.8 Gene Expression by RT‐PCR

Extraction of total RNA from liver tissue was performed with PureLink RNA mini kit following the manufacturer's instructions (Invitrogen, Carlsbad, CA, USA). Transcription was performed to obtain cDNA using the iScript Reverse Transcription Supermix for RT‐qPCR (BioRad, São Paulo, SP, Brazil). Quantitative PCR was performed using the StepOnePlus System (Thermo Fisher Scientific, MA, USA) with Fast SYBR Green Master Mix. Gene expression of carnitine palmitoyl transferase 1 (Cpt1), peroxisome proliferator‐activated receptor alpha (Ppar‐α) and proliferator‐activated receptor gamma coactivator 1‐alpha (Pgc1‐α) was evaluated. Relative gene expression was calculated using the comparative CT method (2^−ΔΔCT^). The results represent the fold change in expression relative to the control group, normalized to the housekeeping gene glyceraldehyde‐3‐phosphate dehydrogenase (GAPDH). The sequences of primer pairs are listed in Table S3.

### 2.9 Statistical Analysis

Mice were randomly divided into groups in cages of six animals. The normality test verified that the samples have a Gaussian distribution. Statistical comparisons were done using, “one‐way” ANOVA followed by Dunnett post‐test. The sample size was calculated with GPower Software (version 3.1.9.7). To calculate the sample size, we used one‐way ANOVA with three experimental groups, effect size = 0.8, α error = 0.05, and statistical power = 0.8. The total sample size consisted of 18 animals, with six mice in each group. All tests and histology analyses were performed in a blinded manner by investigators. Each experimental group initially comprised six animals (*n* = 6). Due to occasional sample loss during processing, some analyses were conducted with less than six animals per group, as specified in the corresponding figure legends. Outliers were identified and removed using Grubbs' test (*α* = 0.05). All statistical analyses and graphs were generated using GraphPad Prism 8.0 (GraphPad Software, San Diego, CA, USA). Data are expressed as mean ± standard error of the mean (SEM), considering *p* < 0.05 as statistically significant.

## 3 Results

### 3.1 MEVs Protected Alveolar Bone Microarchitecture by Keeping Bone Cell Homeostasis

**FIGURE 1:**
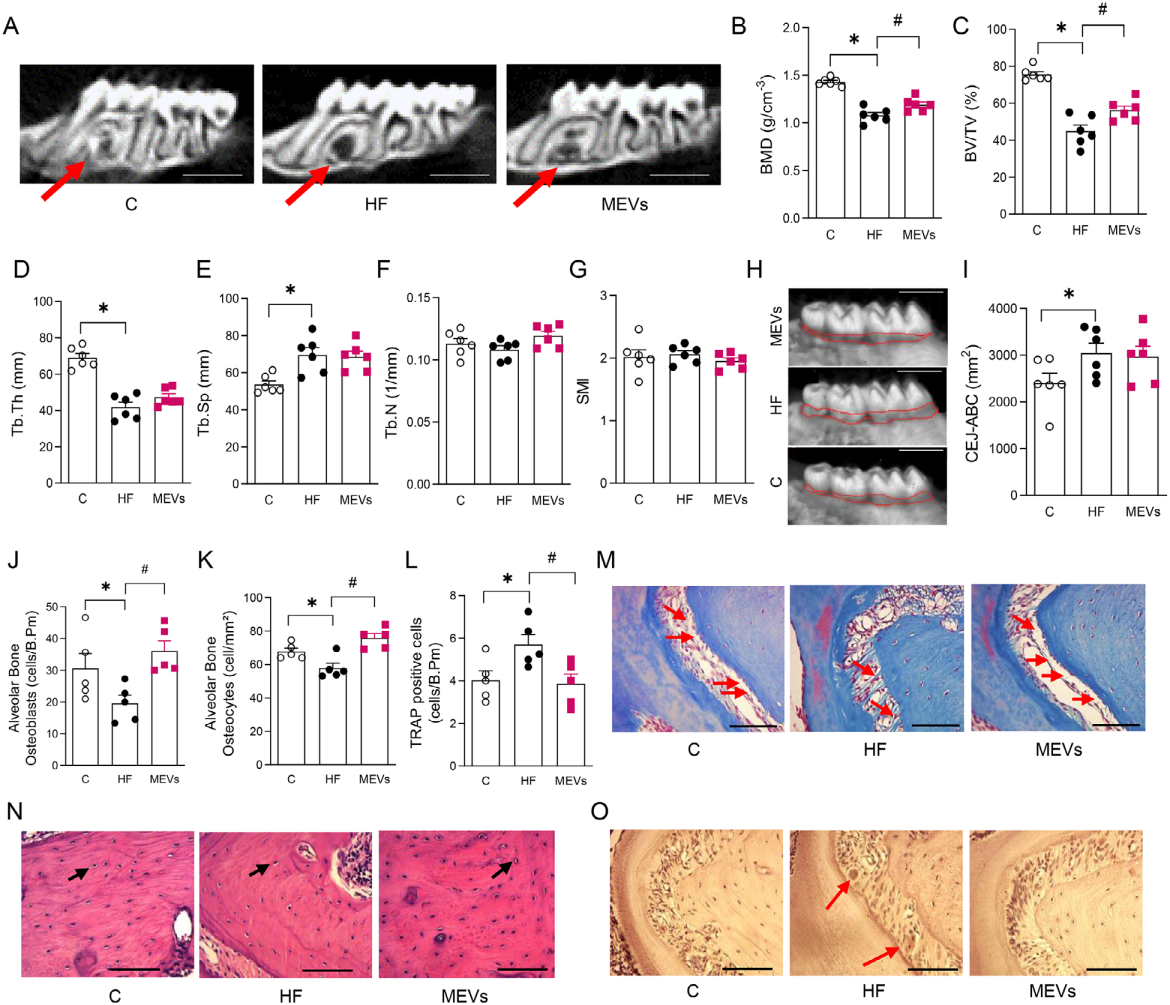
Analysis of the maxillary alveolar bone of mice by computed microtomography and histomorphometry. (A) Representative maxillary image of computed microtomography analysis, (B) bone mineral density (BMD), (C) percent bone volume/tissue volume (BV/TV), (D) trabecular thickness (Tb.Th), (E) separation between trabeculae (Tb.Sp), (F) number of trabeculae (Tb.N), (G) Structure Model Index (SMI), (H) representative CEJ‐ABC image, and (I) cementum‐enamel junction to alveolar bone crest (CEJ‐ABC) (*n* = 6 per group). Micro‐CT scale bars, 25 µm. (J) Osteoblast, (K) osteocyte, and (L) TRAP positive cell counts in alveolar bone by histomorphometry (*n* = 5 per group). Representative image of (M) osteoblasts, (N) osteocytes, and (O) TRAP cells in the alveolar bone (x400, histological scale bars, 100 µm) of mice fed a control (C) diet or a high‐fat (HF) diet for 12 weeks and treated with bovine MEVs in the last 4 weeks. Bars represent mean values ± standard error of the mean. Statistical difference represented by *p < 0.05 ‐ HF versus C and #*p* < 0.05 ‐ HF versus MEVs, one‐way ANOVA, Dunnett posttest for all data.

Analysis of the maxillary alveolar bone microarchitecture demonstrated a reduction in BMD, BV/TV and Tb.Th, and increased Tb.Sp in mice fed HF diet compared with the control group (Figure 1A–E). Additionally, Tb.N and SMI values remained unchanged (Figure 1F,G). The treatment with MEVs led to a significant improvement in BMD and BV/TV compared with non‐treated mice that received the HF diet (Figures 1A–C), although no changes were observed in Tb.Th, Tb.Sp, Tb.N, and SMI (Figures 1D–G). ABC‐CEJ was also augmented in mice fed a HF diet, but no significant impact of MEV treatment was observed (Figure 1H,I). The analysis of tooth roots also did not show significant differences among the groups (Figure S1).

The histomorphometric analysis of the maxilla demonstrated that the mice fed the HF diet had reduced osteoblasts and osteocytes in the alveolar bone compared with the controls (Figure 1J,K,M,N). In contrast, MEV treatment reversed the effect of the HF diet, restoring the number of these cells to a level comparable to the control group (Figure 1J,K,M,N). Consistently, MEV treatment normalized osteoclastic activity (Figure 1L,O).

### 3.2 MEVs Did Not Alter Femoral Bone Loss Induced by the HF Diet

**FIGURE 2:**
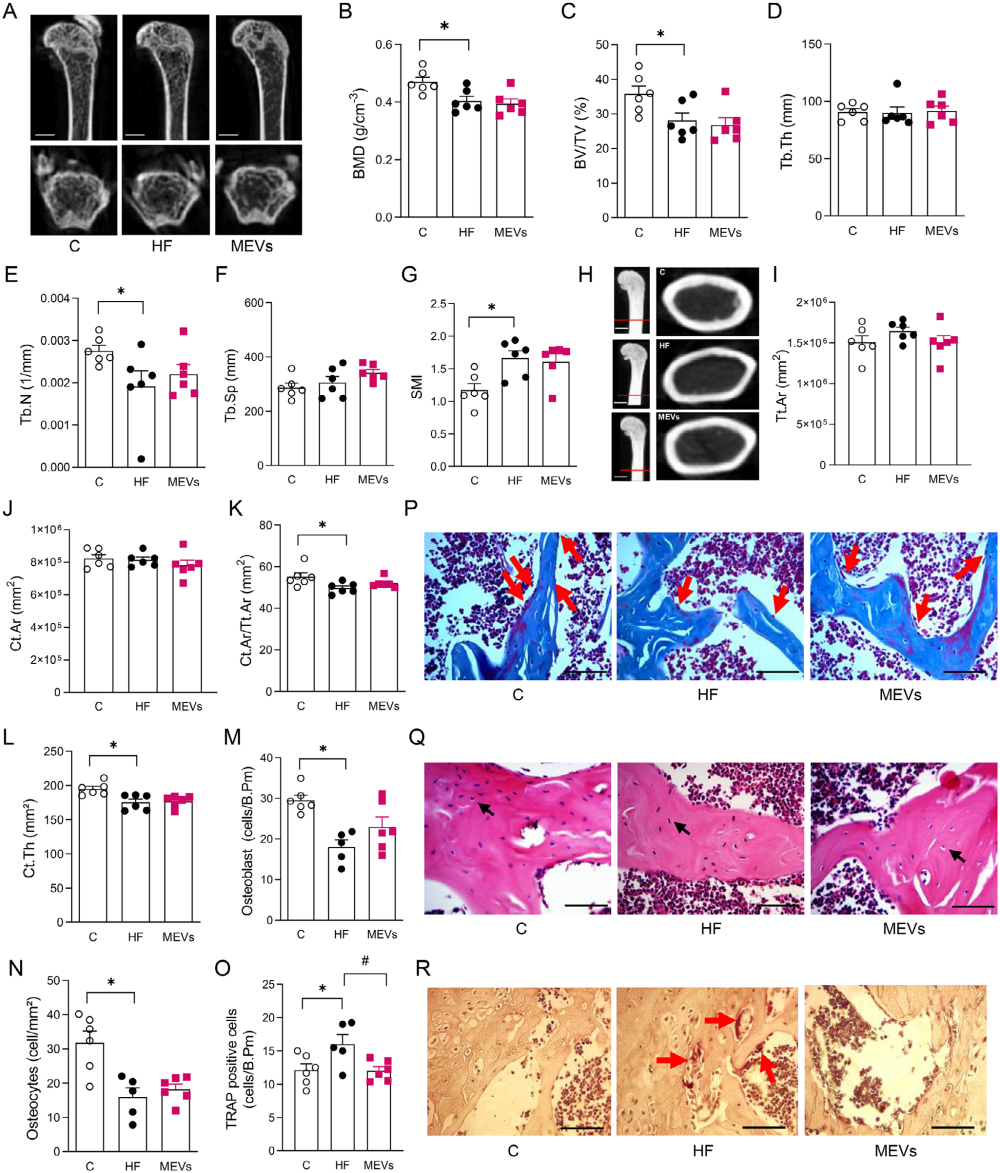
Analysis of the femur of mice by computed microtomography and histomorphometry. (A) Representative femur image of computed microtomography analysis, (B) bone mineral density (BMD), (C) percent bone volume/tissue volume (BV/TV), (D) trabecular thickness (Tb.Th), (E) number of trabeculae (Tb.N), (F) separation between trabeculae (Tb.Sp), and (G) structure Model Index (SMI). Micro‐CT scale bars, 10 µm. (H) Representative cortical femur image of computed microtomography analysis, (I) total cross‐sectional area inside the periosteal envelope (Tt.Ar), (J) cortical bone area (Ct. Ar.), (K) cortical area fraction (Ct.Ar/Tt.Ar), and (L) average cortical thickness (Ct.Th) (*n* = 6 per group). Micro‐CT scale bars, 5 µm. (M) Osteoblast, (N) osteocyte, and (O) TRAP positive cells in trabecular bone by histomorphometry (*n* = 5–6 per group). Representative image of (P) osteoblasts, (Q) osteocytes, and (R) TRAP positive cells in the trabecular bone (x400, histological scale bars, 100 µm) of mice fed a control (C) diet or a high‐fat (HF) diet for 12 weeks and treated with bovine MEVs in the last 4 weeks. Bars represent mean values ± standard error of the mean. Statistical difference represented by *p < 0.05 ‐ HF versus C and #*p* < 0.05 ‐ HF versus MEVs, one‐way ANOVA, Dunnett posttest for all data.

The trabecular bone microarchitecture of the femur demonstrated a significant reduction in BMD, BV/TV, Tb.N, and SMI in mice fed the HF diet compared with controls, while the parameters of Tb.Th and Tb.Sp were not modified (Figure 2A–G). Treatment with MEVs did not change trabecular bone parameters compared with the HF group. Furthermore, the study of cortical bone demonstrated a reduction in Ct.Ar/Tt.Ar and Ct.Th in the femur of HF mice compared with controls, with no differences observed in the values of Tt.Ar and Ct.Ar (Figure 2H–L). Also, the treatment with MEVs did not alter the cortical bone parameters of the femur (Figure 2H–L).

**FIGURE 3:**
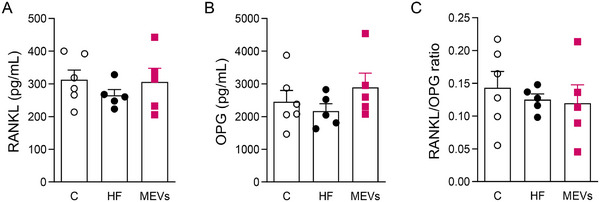
Analysis of systemic bone remodeling markers of mice by ELISA. (A) RANKL, (B) OPG, and (C) RANKL/OPG ratio in serum of mice fed a control (C) diet, or a high‐fat (HF) diet for 12 weeks and treated with bovine MEVs in the last 4 weeks (*n* = 5–6 per group). Bars represent mean values ± standard error of the mean. Statistical difference represented by *p < 0.05 ‐ HF versus C and #*p* < 0.05 ‐ HF versus MEVs, one‐way ANOVA, Dunnett posttest for all data.

Complementarily, the histomorphometric analysis of bone cells in the femur demonstrated a significant reduction in osteoblasts and osteocytes in the trabecular bone of the HF group. Unlikely its effect in maxilla, MEV treatment did not change the number of these cells in the femur (Figure 2M,N,P,Q). However, the treatment normalized the elevated number of TRAP‐positive cells found in the HF diet group (Figure 2O,R). Serological evaluation of RANKL, OPG, and RANKL/OPG markers did not demonstrate significant differences among the groups (Figure 3A–C).

### 3.3 Treatment With MEVs Did Not Significantly Change Adipose Tissue and Systemic Metabolism, but Improved Hepatic Fat Accumulation Induced by HF Diet Consumption

**FIGURE 4:**
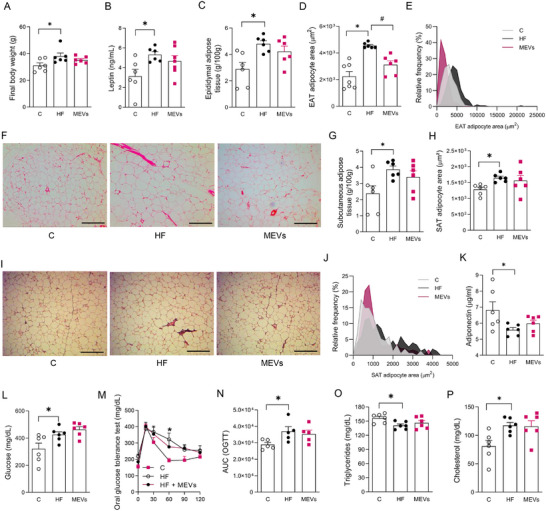
Analysis of weight, body composition, and metabolic alterations of mice. (A) Final weight, (B) serum Leptin by ELISA, (C) weight of epididymal adipose tissue (EAT), (D) EAT adipocyte area by histomorphometry analysis, (E) stratification of the EAT adipocyte area, (F) representative image of EAT adipocyte area (x100), (G) weight of subcutaneous adipose tissue (SAT), (H) SAT adipocyte area by histomorphometry analysis, (I) representative image of SAT adipocyte area (x100), and (J) stratification of the SAT adipocyte area. Histological scale bars, 100 µm. (K) serum Adiponectin by ELISA, (L) serum glucose, (M) oral glucose tolerance test (OGTT), (N) area under the curve (AUC)—OGTT, (O) serum triglycerides, and (P) serum total cholesterol of mice fed a control (C) diet or a high‐fat (HF) diet for 12 weeks and treated with bovine MEVs in the last 4 weeks (*n* = 6 per group). Bars represent mean values ± standard error of the mean. Statistical difference represented by *p < 0.05 ‐ HF versus C and #*p* < 0.05 ‐ HF versus MEVs, one‐way ANOVA, Dunnett posttest for all data.

Animals fed the HF diet demonstrated a significant increase in body weight, associated with higher leptin levels (Figure 4A,B). Accordingly, there was a significant increase in EAT weight and adipocyte size in this tissue compared to the control group (Figure 4C–F). Comparing HF animals with mice treated with MEVs, although no alterations were observed in body weight, leptin levels, and EAT mass (Figures 4A–C), smaller adipocytes were observed in the EAT mean area. This data was consistent with a higher frequency of smaller adipocytes in the MEV‐treated group compared to the untreated HF mice (Figure 4D–F). Also, the study of the SAT of the animals demonstrated a significant increase in weight and area of this tissue in mice fed the HF diet. Still, it was not altered by MEV treatment (Figure 4G–J).

The adiponectin levels were reduced in HF animals (Figure 4K). Concurrently, fasting glycemia increased, which was associated with lower glucose tolerance and dyslipidemia, marked by higher triglyceride and total cholesterol levels in HF mice compared to controls (Figure 4L–P). However, MEV treatment did not significantly alter the evaluated metabolic parameters compared to the HF group (Figure 4K–P).

**FIGURE 5:**
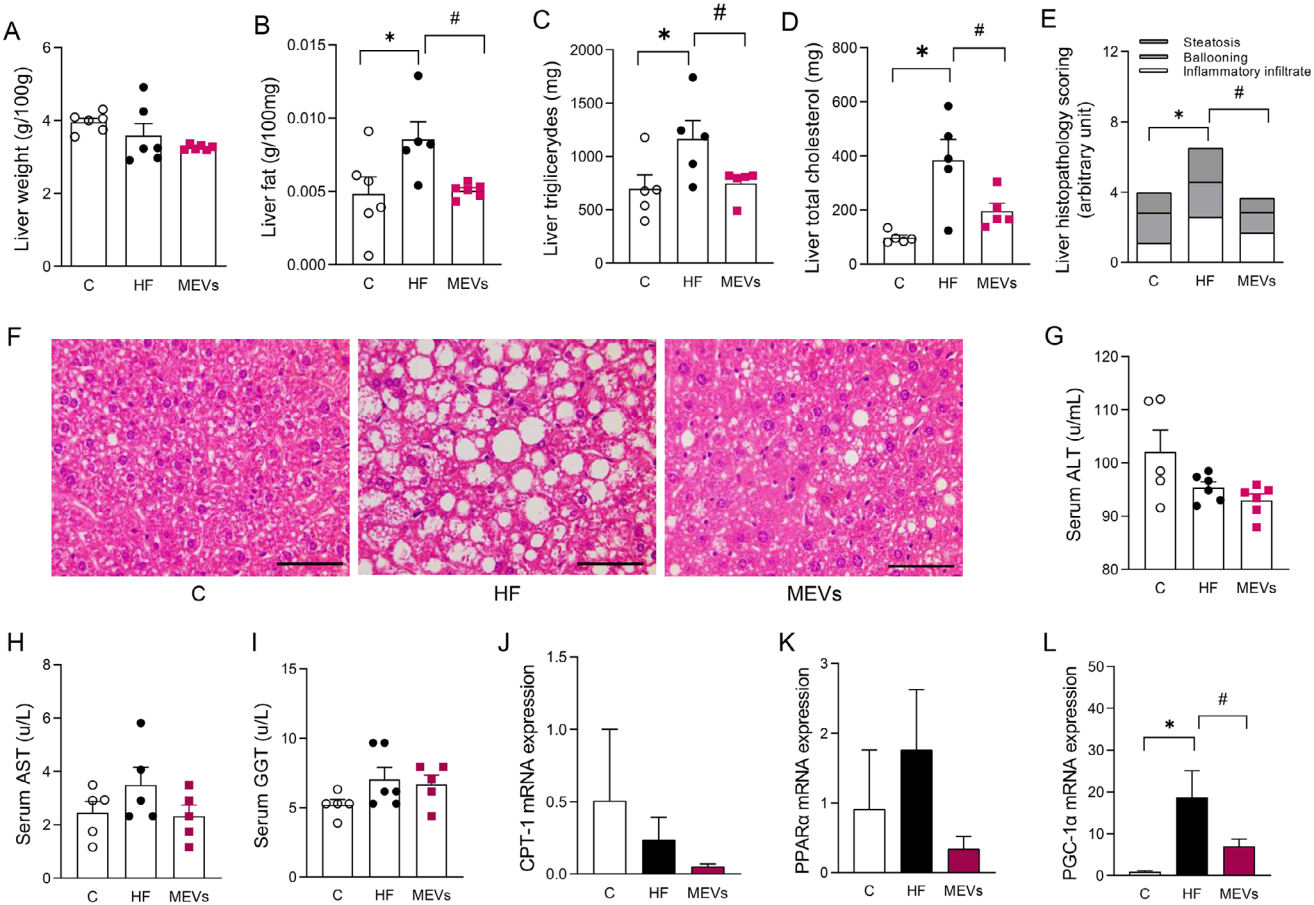
Liver histological and metabolic analysis of mice. (A) Liver weight, (B) total liver fat, (C) liver triglycerides, (D) liver cholesterol (E) liver histopathological score, (F) representative image of the liver histopathological score (×400), (G) serum alanine aminotransferase (ALT), (H) serum aspartate aminotransferase, and (I) serum gamma‐glutamyl transferase (*n* = 5–6 per group). Histological scale bars, 100 µm. (J) Liver Cpt‐1 expression by RT‐PCR, (K) liver Pparα expression by RT‐PCR, and (L) liver Pgc‐1α expression by RT‐PCR of mice fed a control diet (C), or a high‐fat (HF) diet for 12 weeks and treated with bovine MEVs in the last 4 weeks (*n* = 3–6 per group). Bars represent mean values ± standard error of the mean. Statistical difference represented by *p < 0.05 ‐ HF versus C and #*p* < 0.05 ‐ HF versus MEVs, one‐sway ANOVA, Dunnett posttest for all data.

No significant differences in hepatic weight were observed. However, total liver fat, liver triglycerides, and total cholesterol deposition were increased in the tissue of animals fed the HF diet compared with the control group. Interestingly, a clear reversion was seen in MEV‐treated mice that showed a reduction in total fat, triglycerides, and total cholesterol in the liver (Figure 5A–D). Complementary to this, the analysis of the liver histopathological score demonstrated an increase in liver damage in HF animals compared to the control. This damage was substantially reduced in animals treated with MEVs compared to those not treated (HF) (Figure 5E–F). Serum ALT, AST, and GGT analysis did not demonstrate differences between the groups (Figure 5G–I). Hepatic gene expression did not indicate significant differences between Cpt‐1 and Ppar‐α levels. However, it demonstrated an increase in Pgc1‐α in mice fed the HF diet compared to the control (Figure 5K,L). Concomitantly, mice treated with MEVs showed lower expression of this marker (Figure 5L).

## 4 Discussion

We used the treatment with MEVs as a therapeutic strategy for HF diet‐induced bone and metabolic changes derived from obesity. While our previous work showed that MEVs could mitigate systemic bone loss and metabolic dysfunction in mice fed a high‐carbohydrate (HC) diet, a model of moderate obesity [12, 21], the same treatment resulted in only minor improvement in a more severe HF diet model. Nevertheless, MEVs demonstrated a protective effect on alveolar bone microarchitecture, a positive modulation of bone cells, and significantly reduced liver damage, primarily by improving hepatic steatosis.

Our findings show an osteoprotective effect of MEVs in the context of obesity, with a benefit on alveolar bone but a limited impact on the femur. In the maxilla, MEV treatment improved microarchitectural parameters, alongside a positive cellular balance characterized by increased osteoblasts, osteocytes, and reduced osteoclasts. This localized cellular effect may be related to the fact that MEVs have direct contact with the oral mucosa when orally administered and may even interact with the oral microbiota [29, 30]. Given that other particles can be captured through pores or spaces in the periodontal ligament [31], which is in direct contact with the alveolar bone, it is plausible to hypothesize that MEVs could similarly accumulate in this area. This proximity could favor greater accumulation and interaction of the components with the adjacent bone tissue, potentially enhancing their biological effects. This effect could be related to the ease with which MEVs reach the tissues of the oral cavity through the diffusion of fluids and/or nutrients, facilitating cellular communication and the action of their components, such as miRNAs [31]. This hypothesis is supported by the established role of extracellular vesicles as natural drug delivery systems, capable of protecting and transporting bioactive molecules to target tissues [32]. Although a reduction in osteoclast activity was also shown in the femur, this alteration was insufficient to prevent the microarchitectural loss induced by the HF diet. This suggests that MEVs can exert a local cellular effect, but a systemic impact may be diminished in this severe obesity model.

The contrasting outcomes between the alveolar and femoral bone, as well as the limited systemic metabolic effects, are likely due to the more severe nature of the HF diet‐induced obesity compared to our previous high‐carbohydrate model [21]. The inefficiency of MEVs in HF‐diet‐induced obesity can be attributed to changes in the intestinal mucosa and reductions in nutrient absorption [33, 34]. Studies have already demonstrated that MEVs are absorbed through endocytosis by enterocytes via glycoprotein signaling [35, 36], and changes in this microenvironment could hinder this process. Additionally, as MEVs are lipid membrane structures [32, 37], they could be excreted via feces due to the high fat content of the diet, further limiting their systemic bioavailability. Thus, it is plausible that a reduction in systemic effects, including the lack of protection of the femoral bone structure, results from this particle loss.

Interestingly, although MEVs failed to reverse systemic metabolic dysfunction, they significantly mitigated liver damage and hepatic steatosis caused by the HF diet. MEV treatment reduced liver damage in HF‐fed mice associated with a reduction in total fat, triglycerides, and cholesterol deposition. This protective effect aligns with previous studies showing that milk‐derived extracellular vesicles can attenuate hepatic steatosis by modulating lipogenesis and lipolysis [38]. Similarly, MEVs attenuated hepatic fibrosis in vitro in a study by Reif et al., demonstrating a positive effect of these components in the liver [39]. Crucially, a recent study provides a mechanistic explanation for these findings, demonstrating that MEVs cross the intestinal barrier and accumulate significantly in hepatocytes, especially under conditions of metabolic alterations induced by a high‐fat diet, improving dysfunctions such as, inflammation and fibrosis [40]. These findings directly support our results, suggesting that the preferential uptake of MEVs by the liver underlies the observed reduction in hepatic steatosis.

Although we did not observe changes in Pparα and Cpt‐1 expression, MEV treatment reduced Pgc‐1α expression, a coactivator related to the activation of oxidative pathways [41, 42]. This molecule had been elevated by the HF diet, possibly as an unsuccessful adaptive response to the marked fat deposition [43]. We hypothesize that miRNA‐148a, an abundant component of MEV, may contribute to the beneficial effects on lipid metabolism [44, 45]. Notably, Pgc‐1α is a potential target of miRNA‐148a, suggesting that its modulation in response to MEVs could represent a mechanism underlying the observed effects on this molecule [44, 45]. Since the liver is a major site of MEV deposition after absorption [38, 46], we suggest that the intense metabolic stress in the HF model led to preferential uptake and action of MEVs in this organ, mitigating local damage, but simultaneously limiting their systemic availability for the femur. However, more studies are needed to fully understand these mechanisms.

MEV treatment was unable to alter the metabolic parameters and adipose tissue expansion induced by the HF diet. This is in contrast to a previous study from our research group where MEVs improved adiposity, serum glucose, and triglycerides in mice fed a high‐refined carbohydrate (HC) diet, with consequent protection against bone loss [21]. Also, our findings demonstrated no significant differences among the groups for the serological analysis of key bone remodeling markers [47], RANKL, OPG, and their ratio, which may be related to the persistence of systemic metabolic changes even after MEV treatment. The failure of MEVs to reverse these systemic alterations may be due to the more severe metabolic dysfunction of the HF diet compared to the HC diet [21]. We suggest that this severity, combined with factors such as MEV dose, treatment time, and potential changes in intestinal absorption, limited the systemic availability of the components and, consequently, their impact on adipose tissue and systemic metabolism. This localized action may also explain why MEVs were able to protect alveolar bone and mitigate liver damage but failed to reverse femoral bone loss.

Our experimental design included specific methodological choices to balance scientific rigor with animal welfare and physiological relevance. The dosage of 14.3 × 10^6^ particles/mL was selected based on our prior doseresponse findings demonstrating efficacy in modulating bone turnover [19, 20]. We opted for administration via drinking water to mimic physiological intake and maintain continuous contact with the oral mucosa, thereby favoring our hypothesis of a local effect while avoiding the confounding metabolic stress associated with daily gavage. Similarly, group housing with environmental enrichment was maintained to prevent isolation stress. Although these conditions limit individual intake monitoring, the low variability observed across parameters suggests consistent consumption and uniform biological responses. Regarding stability, the solution was refreshed every 2–3 days to minimize degradation. Nevertheless, given that some systemic parameters showed beneficial trends without statistical significance, future protocols should investigate doseresponse curves to determine if higher concentrations could enhance systemic bioavailability.

In conclusion, our study confirms that a high‐fat diet leads to severe metabolic and bone loss in mice. Bovine MEVs were ineffective in reversing systemic bone loss in the femur, but they provided significant localized protective effects on the alveolar bone, modulating cellular homeostasis. Additionally, MEVs contribute to reduced hepatic fat accumulation and are promising in the treatment of hepatic steatosis. However, the action of MEVs on bone loss induced by the HF diet was limited, possibly due to the liver's greater uptake of these components being affected. Complementarily, metabolic changes in diet‐induced obesity may also explain the bone changes found in this model and the failure of MEVs to reverse the pathological bone phenotype. Further studies are needed to gain a deeper understanding of their mechanisms of action and assess their effectiveness for future clinical applications.

## Conflicts of Interest

The authors declare no conflicts of interest.

## Supporting information




**Supporting File**: mnfr70400‐sup‐0001‐SupMat.pdf.

## Data Availability

The data that support the findings of this study are available on request from the corresponding author. The data are not publicly available due to privacy or ethical restrictions.
