# Supplementary material for: Bovine Milk Extracellular Vesicles Modulate Alveolar Bone Microarchitecture and Mitigate Hepatic Steatosis in Obese Mice Fed a High‐Fat Diet
Source: Mol Nutr Food Res. 2026 Feb 5;70(3):e70400. doi: 10.1002/mnfr.70400 (PMC12874502; doi:10.1002/mnfr.70400)
Supplement: Supplementary file 1 — Supporting File: mnfr70400‐sup‐0001‐SupMat.pdf. [file MNFR-70-e70400-s001.pdf]

Supplementary Table 1. Compositions of experimental diets.

Ingredients	Experimental diets	
	**Control AIN-93M (g/kg diet)**	**High fat 45% (g/kg diet)**
Cornstarch	465.692	253.00
Dextrinized cornstarch	155.00	-
Casein	140.00	241.00
Gooseberry syrup	-	181.00
Lard	-	145.00
Sucrose	100.00	-
Fiber	50.00	60.00
Soybean oil	40.00	60.00
Mineral mix	35.00	42.00
Vitamin mix	10.00	12.00
Methionine	-	4.00
Choline bitartrate	2.50	2.00
L-Cystine	1.80	-
Butylated hydroxytoluene	0.008	0.04

Supplementary Table 2. Nutritional information of experimental diets.

Diet composition (%)		
**Substrates**	**Control (AIN-93M)**	**HF (45%)**
Carbohydrates	75.9	39.0
Fats	10.3	44.0
Proteins	13.7	17.0
Energy Density	3.6	6.2

Supplementary Table 3. Real-time PCR primer sequence.

Gene	Accession Nº	Forward	Reverse	Amplicon size (bp)
Cpt-1	NM_009948.2	5'-TCCCAGGCAAAGAGACAGACTTGC-3'	5'-GCAGGCGCGAGCCCTCATAG-3'	111
Ppar-α	NM_011144.6	5'-TTGTGGCTGGTCAAGTTCGGG-3'	5'-CCACAGAGCGCTAAGCTGTGA-3'	57
Pgc1α	NM_001402987.1	5'-GGAGCCGTGACCACTGACA-3'	5'-TGGTTTGCTGCATGGTTCTG-3'	176
Gapdh	NM_008084.4	5'-ACGGCCGCATCTTCTTGTGCA-3'	5'-CGCCCAAATCCGTTCACACCGA-3'	78

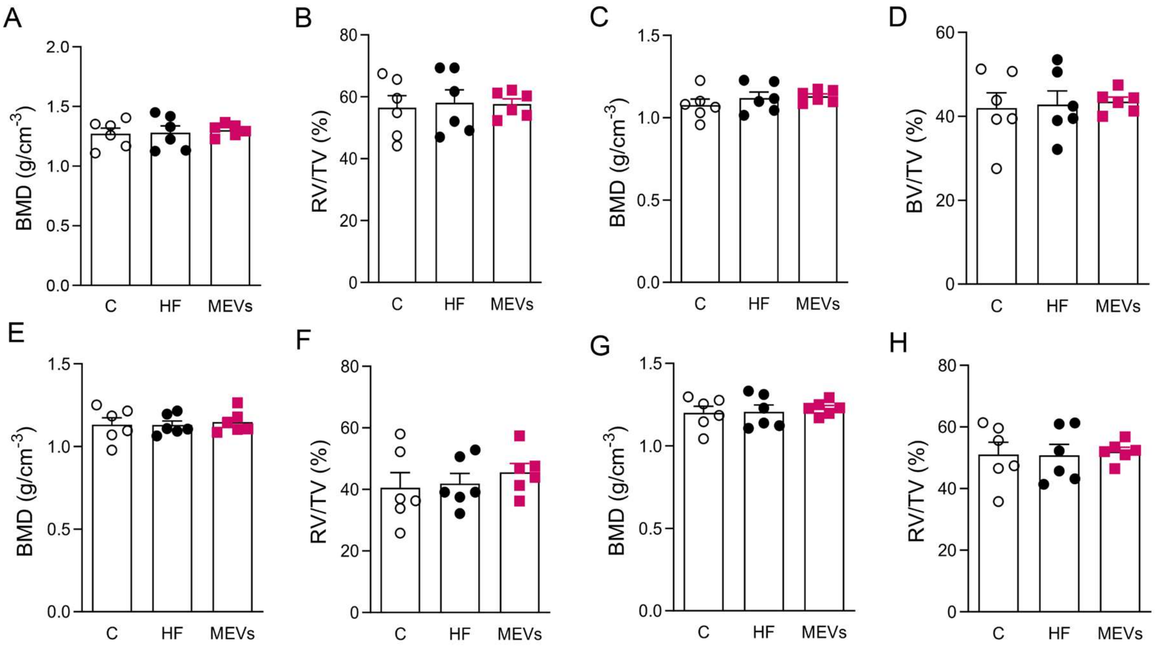

Supplementary Figure 1: Analysis of the maxillary roots of mice by computed microtomography. (A) Bone mineral density (BMD) and (B) percent bone volume/tissue volume (BV/TV) of mesiobuccal root. (C) BMD and (D) BV/TV of palatal root, (E) BMD and (F) BV/TV of distobuccal root, (G) BMD and (H) BV/TV of all maxillary roots of mice fed a control (C) diet or high-fat (HF) diet for 12 weeks and treated with bovine milk extracellular vesicles (MEVs) in the last 4 weeks (n = 6 per group). Bars represent mean values ± standard error of the mean. Statistical difference represented by *$p < 0.05$ - HF vs. C and #$p < 0.05$ - HF vs. MEV, one-way ANOVA, Dunnett posttest for all data.
